# Editorial: The network theory of epilepsy at twenty

**DOI:** 10.3389/fnetp.2026.1973521

**Published:** 2026-09-15

**Authors:** Hitten P. Zaveri, Klaus Lehnertz, Dennis D. Spencer

**Affiliations:** 1 Department of Neurology, Yale University, New Haven, CT, United States; 2 Department of Epileptology, University of Bonn Medical Centre, Bonn, Germany; 3 Helmholtz Institute for Radiation and Nuclear Physics, University of Bonn, Bonn, Germany; 4 Interdisciplinary Center for Complex Systems, University of Bonn, Bonn, Germany; 5 Department of Neurosurgery, Yale University, New Haven, CT, United States

**Keywords:** biomarkers, complex networks, EEG, epilepsy, network theory of epilepsy, seizures

## Introduction

In 2002, Susan Spencer proposed that a seizure should be understood not as the product of a single aberrant focus but as the expression of a functionally and anatomically connected set of cortical and subcortical structures, in which vulnerability to seizure activity in any one part is shaped by activity everywhere else in the network (Spencer, 2002). The idea proved compelling enough to become one of the field’s two dominant accounts of how seizures arise, but not compelling enough to settle into consensus: by 2019, the International Conference for Technology and Analysis of Seizures (ICTALS) convened formal debate sessions on six unresolved questions, among them whether the epileptic network is a distinct pathological entity or an existing physiological network gone awry, whether the network must be fully defined before it can be controlled, and whether controlling seizures is the same as controlling the network (Zaveri et al., 2020). A later perspective piece from this journal catalogued the field’s open challenges and called for studies to address them (Sinha et al., 2022). This Research Topic of *Frontiers in Network Physiology–Networks in the Brain System* section, marking more than 20 years since Spencer’s original statement, gathers seventeen contributions that amount, in aggregate, to a partial answer to that call, and the clearest progress visible across them is not on the theory’s founding claim, which these contributions do not dispute, but on the practical machinery of finding, characterizing and treating the network in an individual patient.

**FIGURE 1:**
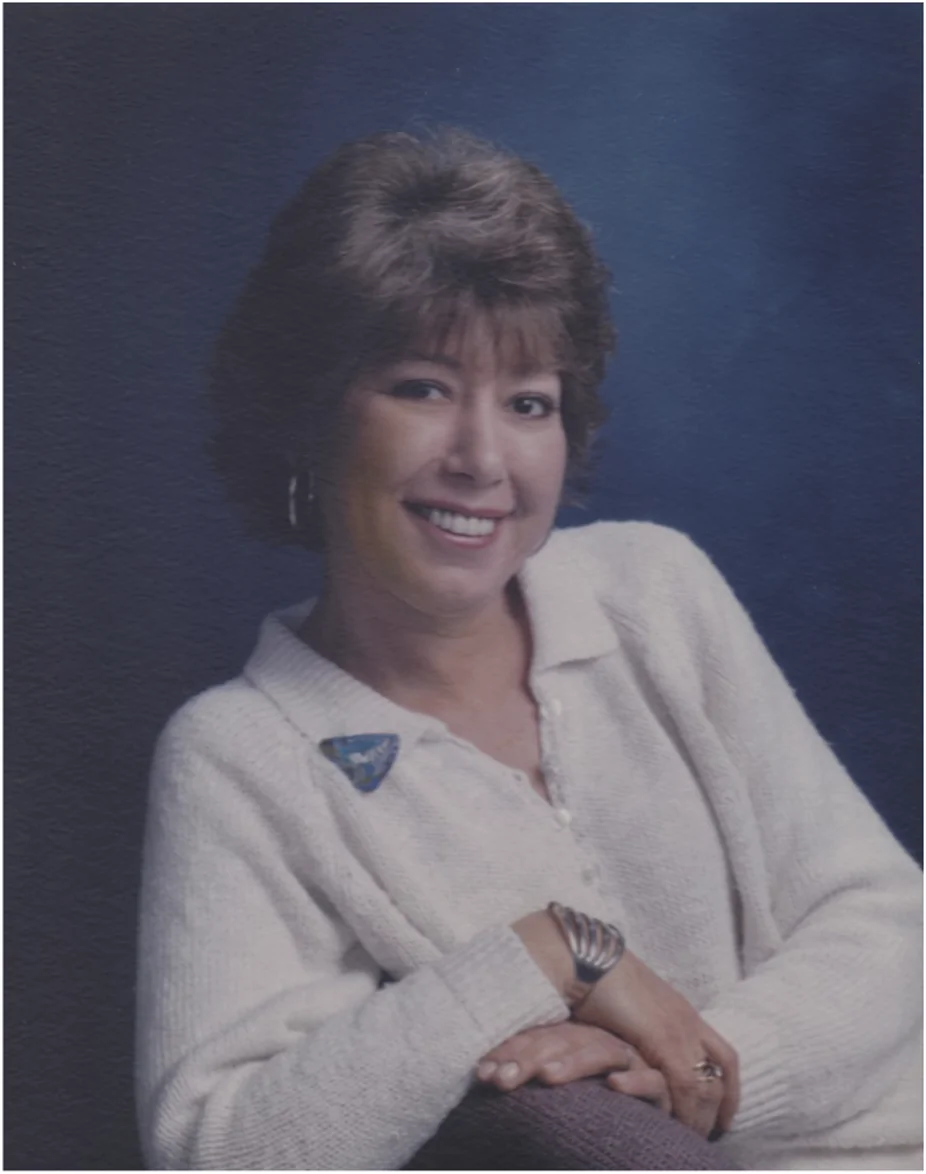
Susan Spencer, MD.

## Biomarkers of the network

The largest cluster of papers in this Research Topic addresses exactly that problem, and it is where the Research Topic’s empirical weight sits. Herlopians’s review reframes the high-frequency oscillation (HFO) itself, reading the evidence as showing that HFOs are not fixed to a single site but propagate along sequential pathways from an onset contact to spread contacts across cortex within milliseconds: a discrete expression of the network they traverse rather than a local event, with the full propagation zone predicting the resection zone more reliably than any single high-rate contact. Pinto-Orellana and Lopour build on this premise, quantifying how consistently each channel’s HFO activity synchronizes with the rest of the network via a channel-level connectivity dispersion metric; tested on 89 and 15 patients from independent centers, seizure-onset-zone channels showed lower, not higher, dispersion than surrounding tissue, with the difference tracking surgical outcome. Joshi et al. propose infraslow (below 0.2 Hz) envelope correlations as a candidate edge-forming process, estimating them from 3-min recording windows and showing that significantly nonzero correlation persists across time lags of up to about a minute, distinct from the millisecond-to-second range most connectivity measures operate on; correlations rose modestly after medication taper, consistent with heightened seizure vulnerability. Kashyap et al. compare five interictal EEG features for tracking the circadian and multidien cycles that modulate seizure risk, finding that network-derived measures phase-lock to seizures more tightly than interictal epileptiform discharge rate and remain more robust when subsampled, with implications for implantable seizure-forecasting devices. Zhai et al. developed patient-specific dynamical network models from interictal intracranial EEG to simulate stimulation response *in silico*, discriminating epileptogenic zone (EZ) from non-EZ tissue with an AUC of 0.95 across 69 patients from five centers; non-EZ channels also showed elevated excitability in patients with successful surgical outcomes, a pattern absent in failures. Roy et al. show that the earlier published (Gunnarsdottir et al., 2022) source-sink index’s steady-state behavior can be derived as the leading eigenvector of the state-transition matrix of a dynamical network model, predicting surgical success with roughly 72% accuracy from only 5 min of interictal recording across 65 patients at six centers. Jahani et al.’s preictal directed-connectivity analysis, applied to a multicenter intracranial EEG dataset of 243 seizures from 61 patients, discriminated the seizure onset zone with a higher AUC than connectivity during the seizure, but only once patients were regrouped by whether their dominant preictal pattern was inflow toward, or outflow from, the seizure onset zone. Read as a set, these seven papers converge less on a single biomarker than on a common signature: the seizure onset zone is consistently more legible through its relationship to the surrounding network than through any property intrinsic to the tissue itself, evidence for the network account of epileptogenicity that Spencer’s original theory (Spencer, 2002) proposed only in outline.

## Mechanism and substrate

Two papers push toward mechanism at a level the biomarker studies cannot reach directly, and both speak to the ICTALS question of whether the epileptic network is pathological or physiological. Bower’s review argues for the latter reading, showing that the specific ensembles of single neurons active at seizure onset are selectively reactivated during post-seizure slow-wave sleep but not wakefulness, a pattern the author terms seizure-related consolidation, borrowed directly from the mechanisms of ordinary memory formation; on this account epileptogenesis co-opts a physiological process rather than representing a wholly separate pathology. Boddeti et al. reach a compatible conclusion from an *in vitro* microelectrode-array model of neuron-glia cultures, in which chronic exposure to 4-aminopyridine, a potassium-channel blocker, produces functional connectivity that grows not merely stronger but bimodal in distribution within 2 days of otherwise-naive culture (Cohen’s d=1.43 ): evidence that ordinary circuitry is sufficient substrate for network pathology to emerge under sustained hyperexcitability.

## The network beyond the seizure onset zone

Several papers document that the practical consequences of network involvement extend well past the tissue a surgeon would resect, bearing on the ICTALS question of whether controlling seizures is the same as controlling the network. Wheeler et al. describe psychosis that emerged during continuous high-frequency anterior thalamic stimulation in two patients with mesial temporal lobe epilepsy, even as stimulation suppressed seizures substantially from baseline, suggesting a network disrupted enough to stop seizures can still misbehave in the memory, mood, and sleep domains the same circuitry ordinarily supports. Switching to a duty-cycle stimulation pattern resolved the psychiatric symptoms without sacrificing seizure control, suggesting the two goals are separable in practice even if the seizures and psychosis result from the same network. Murray et al. supply a related finding for cognition: using stereo EEG in 48 surgical patients, they show that naming, fluency, and recognition-memory deficits track the extent and direction of the interictal “irritative” network rather than the seizure onset zone, that is, a network’s cognitive footprint can be substantially larger than its seizure-generating footprint. Cui et al. offer a more optimistic instance of the same question: in two patients whose scalp EEG showed features of a generalized epileptic encephalopathy, focal resection of a single driving node eliminated every seizure type and the generalized-appearing EEG features resolved completely, suggesting that removing the right node can collapse far more of the network’s pathological behavior than its apparent size would predict.

## Infrastructure and translation

Two further contributions look past biomarker development toward what the field will need to act on it. Langbein et al. review current surgical and neuromodulatory strategies through the lens of network theory, and their central observation is sobering: more than half of patients continue to experience refractory seizures within a year of surgery, and they argue that closing this gap will require shifting surgical targeting from focus-removal toward epileptic-network constituent removal (Lehnertz et al., 2023). Collins et al. address a different infrastructure gap, contributing the Yale Brain Atlas, a 690-parcel, neuroanatomical landmark based open-source atlas integrating white-matter connectomes and cortical thickness data from Human Connectome Project subjects with resting-state and meta-analytic functional data, explicitly positioned to standardize neuroanatomy and aid communication, intracranial electrode localization, multimodal data fusion, and decision making for epilepsy surgery.

## Revisiting the network theory

Two contributions engage the theory at the level of its foundational claims and are worth situating last precisely because they attempt to answer the ICTALS debate questions. Bröhl et al. survey the conceptual architecture of the time-evolving epileptic network across the full span of timescales the ICTALS debate raised (from seconds to years) and report a finding that complicates any simple account of what the network is: the vertices that turn out to be most central during a seizure are often not in the clinically defined seizure onset zone but in tissue considered unaffected by the pathology, suggesting the field has sometimes been defining, and attempting to control, the wrong part of the network entirely. Zaveri et al. close the Research Topic with a hypothesis of what the network’s nodes actually are, proposing from their own background intracranial EEG studies that the network reduces, in the main, to two nodes (the seizure onset area and the thalamus) with a low-frequency thalamocortical signature governing seizure initiation and a higher-frequency cortico-cortical signature governing spread. A companion analysis of local connectivity by Zaveri et al. supplies empirical support, in the form of a distance-graded connectivity profile, peaking not at the seizure onset area itself but in a rim one to 5 cm away, that separates surgical outcomes with high accuracy. Whether this parsimonious two-node account can be squared with Bröhl et al.’s more distributed picture, or with the network’s reach into cognition and psychiatry documented elsewhere in this Research Topic, is left substantially unresolved.

## Looking forward

The clearest progress in this Research Topic lies less in resolving whether epilepsy is a network disease, a premise these contributions share, than in the practical, patient-specific machinery for locating that network, and in the growing evidence that surgical failure looks less like mislocalization than like incomplete treatment of a network whose true extent these methods are increasingly able to estimate. That finding, more than any resolution of the 2019 debates (Zaveri et al., 2020), is this Research Topic’s real contribution, and it is also the one with the least established path into changed practice. The task ahead is to close the gap between algorithms that can estimate a network’s extent and a surgical and neuromodulatory practice that has yet to be redesigned around that estimate.
